# Sex and hand differences in circadian wrist activity are independent from sex and hand differences in 2D:4D

**DOI:** 10.1186/1740-3391-7-13

**Published:** 2009-10-29

**Authors:** Camille Reuter, Denise B McQuade

**Affiliations:** 1 Department of Biology, Skidmore College, Saratoga Springs, NY 12866 USA

## Abstract

**Background:**

We investigated the relationship between patterns of sex and hand differences in circadian wrist activity and digit ratio, a marker for prenatal androgen exposure. If the contribution of prenatal androgen exposure to sex differences in digit ratio underlies sex differences in circadian wrist activity, we predict that patterns of wrist activity will be correlated with digit ratio.

**Methods:**

Bilateral wrist activity of male and female college students was measured for three consecutive days. Digit ratio was obtained from photocopy measurements of the second and fourth fingers of each subject.

**Results:**

Males had lower digit ratios with more pronounced differences on the right hand. Female acrophase occurred earlier than male acrophase. There was more activity in the right hand and right hand activity peaked before the left. Digit ratio was not correlated with any measure of wrist activity. An analysis of activity by age revealed that younger female students exhibited more male-like activity patterns.

**Conclusion:**

Sex and hand differences for digit ratio and acrophase replicated previous findings. The lack of correlation between digit ratio and patterns of wrist activity suggests that sexually dimorphic circadian activity develops independently from the mechanisms of hormone exposure that cause sex differences in digit ratio.

## Background

The main circadian pacemaker responsible for maintaining the sleep/wake cycle in mammals is located in two bilaterally symmetrical suprachiasmatic nuclei (SCN) [1]. Each SCN comprises its own oscillator [2,3], but the two structures are thought to work in tandem to generate a single rhythm. However, when both sides of the body are monitored simultaneously (such as with wrist actigraphy), the rhythms for each side vary slightly [2] and the activity of the dominant hand has been shown to peak before the non-dominant hand [2,4]. There are also sexually dimorphic activity patterns, such that females reach acrophase earlier in the day than males [5] and female chronotype is considered to be morning-type while males are deemed evening-types [6,7]. Cerebral dominance may be readily assessed, but how patterns of differences in anatomical and functional asymmetries are correlated to cerebral dominance are not as straightforward [8].

While sex, hand and handedness all appear to influence circadian regulation of activity patterns, their relative contributions remain unknown. The primary goal of the present study was to advance our understanding of the mechanisms which regulate bilateral circadian rhythms by investigating the basis for sex differences in activity patterns. More specifically, this study addressed the role of gonadal hormones assayed indirectly by analysis of digit ratios in the regulation of circadian activity, assessed with wrist activity monitors.

It is well documented that circulating hormones can affect circadian rhythms; for example, estrogen has been shown to shorten circadian period and advance phase in hamsters [9]. Gonadal hormones can also modulate circadian activity, as in mice [10] and *Octodon degus *[11]. There is direct evidence of sex hormones influencing SCN function: estrogen and progesterone receptors are present in the human SCN but females have more estrogen receptors in the SCN than males [12]. Androgen receptors have been localized in the core of the mouse SCN and have greater expression in males than females [10]. Furthermore, gonadectomy alters male mouse circadian activity, which can be restored with testosterone treatment [10]. Thus, the sexually dimorphic combinations of steroid receptors in the SCN could account for sexually dimorphic patterns of circadian activity.

Although circulating hormones can directly affect circadian activity, the role of prenatal hormone exposure on the development of sexually dimorphic activity patterns has not been fully investigated. One non-invasive approach to assessing prenatal exposure to hormones is the use of digit ratios, a measure of the relative lengths of the second digit (most often a finger) to the fourth (2D:4D) which is generally lower in males than females [13,14]. The sex difference is subtle with overlapping ranges for males and females [13,15], and the difference is more pronounced on the right hand than the left [15-17]. Increasingly in the past decade, a number of researchers have explored the relationship of digit ratio, a sexually dimorphic anatomical trait, with other morphological, physiological, and behavioral traits [18-23].

Evidence from multiple lines of research indicates that digit ratio is correlated with prenatal androgen exposure: for example, (i) the sexual dimorphism in digit ratio is seen by the age of two and is relatively stable through puberty, especially in the right hand [14,24]; (ii) 2D:4D is sexually dimorphic in children from a wide ethnic range [25]; (iii) children with congenital adrenal hyperplasia (CAH), who are exposed to high androgen prenatally, have lower 2D:4D (are more masculine) than healthy controls [26]; (iv) low 2D:4D is related to a polymorphism in the androgen receptor that increases the sensitivity to testosterone [27]; (v) females from opposite sex twins have lower 2D:4D than females from same sex twins [28]; (vi) children over age one produce very low levels of sex hormones (until puberty), yet the sex difference in digit ratio is apparent and constant at young ages, suggesting 2D:4D is established during prenatal hormone exposure in utero [14,25]. In fact, the use of digit ratio as a putative marker for prenatal androgen exposure has become routine [18,29,30] since direct measurement of prenatal hormone levels is not feasible in humans [13].

Despite the fact that there are similar patterns of sex and hand differences in digit ratio and circadian wrist activity, the relationship between the two components has never been examined. This study compares the two measures in male and female college students. It is reasonable to hypothesize that prenatal androgen exposure may lead to sex differences in circadian rhythms because: (i) many sexual dimorphisms such as personality traits, sexual orientation, and disease predisposition are linked to prenatal androgen exposure [13,18,22] and (ii) it is already known that the same hormones circulating in adulthood significantly alter circadian activity. If the contribution of prenatal androgen exposure to the development of digit ratio also affects SCN development, we predicted that patterns of circadian activity would be correlated with 2D:4D. In order to eliminate brain lateralization as a variable, only right-handed subjects were included in the study.

## Methods

### Subjects

Ninety right-handed students (45 female) from Skidmore College, New York ranging from 16 to 22 yr (mean = 20.04 ± 0.16) participated in the study during the summer and fall 2008 terms. Subjects were recruited by undergraduate researchers and received no compensation for their participation. Approval for the study was granted by the local Institutional Review Board prior to the collection of data.

### Experimental Procedure

#### Digit Ratios

Digit ratio was obtained through photocopy, using techniques that minimize measurement error [15,31]. A fine-tipped black permanent marker was used to mark the most proximal crease at the base of the second and fourth digits prior to photocopy and two images each of the left and right hands were taken. One hand at a time was placed flat on the glass of the photocopier in a relaxed position (fingers not pushed together or splayed apart) with light pressure applied to the hand. The angle of the arm in relation to the photocopier was consistent at 45 degrees through the use of an angle template. Hands were completely removed from the glass in between copies.

One of two trained researchers measured the length of the digits with a plastic ruler to the nearest 0.05 cm, from the middle of the proximal crease to the fingertip. The lengths of the second (2D) and fourth digits (4D) were averaged for each hand and digit ratio (2D:4D) was calculated separately for each hand by dividing average 2D by average 4D. Asymmetry (D_R-L_) was calculated by subtracting left 2D:4D from right 2D:4D.

Inter-rater reliability of photocopy measurements was assessed using absolute value intra-class correlation coefficients (ICCs). ICCs were computed as r_1 _values (JMP version 5.1, division of Statistical Analysis Systems, SAS Institute, Cary, NC, USA) as:



where MS is mean squares. For right 2D:4D, r_1 _= 0.988 and for left 2D:4D, r_1 _= 0.984.

#### Wrist Activity

Participants wore a wrist activity monitor on each wrist (Actiwatches: Minimitter, Inc., Bend, OR, USA) continuously from Monday to Friday. The Healy Hand Preference questionnaire (HHP) [32] was administered on the Friday of test week to confirm right-handedness. Scores indicate hand preference and can range from 1 (extreme right-handed) to 5 (extreme left-handed). All participants were confirmed as right-handed according to the HHP; males (1.64 ± 0.05) and females (1.66 ± 0.05) displayed the same degree of handedness (p = 0.779). Participants also completed the Horne-Ostberg questionnaire [33] to evaluate morning-evening tendencies (ME) for preferred time of activity.

#### Statistical Analysis

Rhythmwatch Reader and software (Minimitter, Inc.) were used to analyze data from the actiwatches. Amount of activity was recorded over 10-minute intervals throughout data collection. Only wrist activity data from Tuesday to Thursday was included in the analysis to avoid weekend effects and allow for acclimation. Sunrise and sunset times in Saratoga Springs, NY, USA for the Wednesday during the test week were used to define light versus dark photoperiod phases. Mean activity and acrophase were recorded for each hand. Statistical Analysis Systems (SAS, SAS Institute, Cary, NC, USA) was used to calculate one-factor ANOVAs to test for the effect of sex on digit ratio and activity. Two-factor ANOVAs tested for the effects of sex and age. Paired t-tests were used to evaluate differences between hands. Cohen's *d *was calculated to compare the size of the sex effect for digit ratio variables [34]. Pearson's correlations tested the association of digit ratio and digit length with activity variables. Spearman's rank order correlations were also run; results were similar to results from Pearson's correlations and are not shown. Means are reported +/- standard error of the mean (SEM).

## Results

### Digit Ratio

Significant sex differences were found with males having lower 2D:4D than females (Table 1). Sex differences for 2D:4D were greater in the right hand (p = 0.01) and did not achieve statistical significance on the left (p = 0.10), principally due to the lower digit ratio of 0.968 for females on the left vs. 0.977 on the right. There were no statistically significant sex differences in D_R-L _although there was a small Cohen's d effect for both D_R-L _and L2D:4D. Not surprisingly, male 2D and 4D were significantly longer than female 2D and 4D for each hand (p < 0.0001).

**Table 1:** Mean (± standard error) results for digit ratio and digit length (cm) for all 90 participants.

	**Males**	**Females**	***P***	***d***
**R2D:4D**	0.959 ± 0.004	0.977 ± 0.005	0.01	-0.59
**L2D:4D**	0.957 ± 0.004	0.968 ± 0.005	0.10	-0.36
**D_R-L_**	0.002 ± 0.003	0.008 ± 0.004	0.17	-0.29
**R2D**	7.44 ± 0.07	6.86 ± 0.06	<0.0001	1.34
**L2D**	7.41 ± 0.07	6.82 ± 0.05	<0.0001	1.48
**R4D**	7.76 ± 0.07	7.02 ± 0.06	<0.0001	1.71
**L4D**	7.75 ± 0.07	7.05 ± 0.06	<0.0001	1.63

*P*-values are reported from an analysis of variance by sex. Cohen's *d *measures the effect size for sex differences.

### Circadian Wrist Activity and Activity Preferences

**Figure 1:**
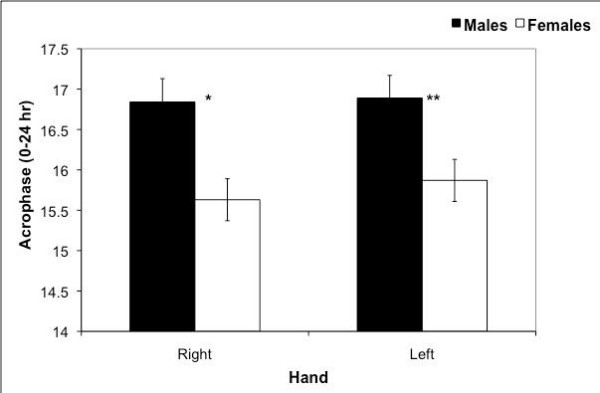
**Acrophase for Right and Left Hands of Male vs. Female Subjects**. Mean (± standard error) acrophase for right and left hands of males and females across 3 days. Females peaked earlier in the day than males in the right (*p = 0.002) and left (**p = 0.008) hands. The right hand peaked before the left in combined subjects (paired t-test; p < 0.01).

No sex differences were seen in mean activity (male 2835 ± 139 and female 2872 ± 112 counts per 10-min interval), amount of right hand (male 2926 ± 133; female 3019 ± 128) or left hand activity (male 2744 ± 179; female 2725 ± 106), or asymmetry of hand activity (the difference between right and left activity; male 182 ± 149; female 293 ± 75). However, significant sex and hand differences were found for acrophase. Females showed mean peak activity 1.12 hours earlier in the day than males (p = 0.004); the sex difference was present for both right (p = 0.002) and left hands (p = 0.008) as shown in Figure 1. The dominant (right) hand peaked before the left (paired t-test; p < 0.01) for combined subjects. Combined subjects also used their right hands significantly more than the left (paired t-test; p < 0.01). Activity for the right hand averaged 2973 ± 92 counts per 10-min interval while activity on the left averaged 2735 ± 104 for a difference of 8.3% more activity on the right.

**Figure 2:**
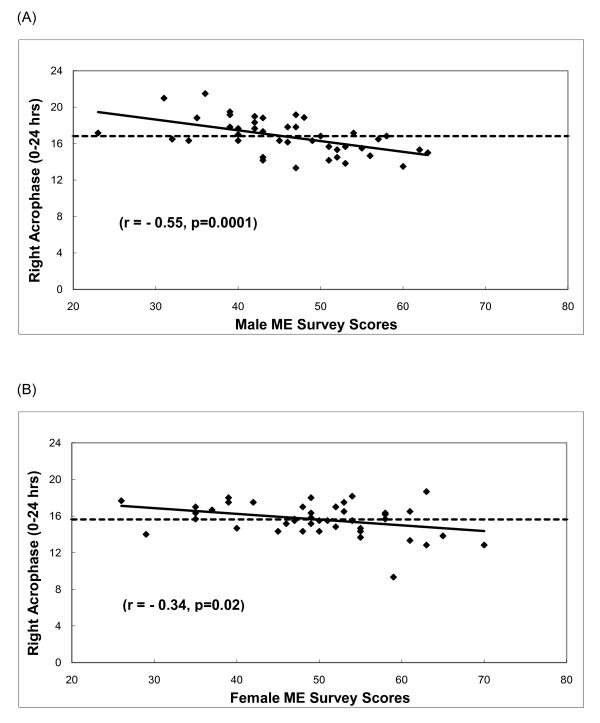
**Correlation Between Male and Female ME Survey Scores and Right Hand Acrophase**. Correlation between Morning-evening activity preference (ME Survey) and acrophase. ME survey scores correlated with right hand acrophase (shown) in males (A, Pearson's r = -0.55, p = 0.0001) and females (B, Pearson's r = -0.34, p = 0.02); ME survey scores also correlated with left hand acrophase (not shown) in males (Pearson's r = -0.56, p < 0.0001) and showed a correlation trend in females (Pearson's r = -0.25, p = 0.09). The negative correlation indicates that the higher the ME score, the earlier the acrophase is reached (indicating more morning-type tendencies). Mean right acrophase is denoted by a dashed line.

The significant sex differences in ME survey results were in accordance with sex differences for circadian patterns of activity: females identified themselves as more morning type (49.87 ± 1.42) while males were more evening type (45.27 ± 1.32; p = 0.020). Furthermore, male ME survey scores were highly correlated with both right hand peak activity (r = -0.55; p = 0.0001) and left hand peak activity (r = -0.56; p < 0.0001). For females, only right hand peak activity correlated with ME survey score (r = -0.34; p = 0.022). Figure 2 displays the correlation for right acrophase with male (A) and female (B) ME survey scores.

### Correlation of Digit Ratio with Measures of Circadian Wrist Activity and Handedness

For all participants, there was a robust lack of correlation between all measures of digit ratio and measures of circadian activity for both hands (Table 2). There was no correlation of 2D:4D with asymmetry in circadian activity (activity_R-L_, acrophase_R-L_). 2D:4D was not correlated with HHP or ME, i.e. low scores for 2D:4D did not predict higher HHP (less right-handed) or more eveningness. Thus, although males had lower 2D:4D than females, and male acrophase was later than female acrophase, low 2D:4D scores in males was not predictive of later peak activity within males and high 2D:4D scores in females was not predictive of earlier peak activity within females.

**Table 2:** Pearson's correlation coefficient between digit ratio and measures of circadian wrist activity.

	**Males**		**Females**	
	**R2D:4D**	**L2D:4D**	**R2D:4D**	**L2D:4D**

**Mean Acrophase**	0.08	-0.05	-0.06	-0.04
**R Acrophase**	0.06	-0.06	-0.10	-0.04
**L Acrophase**	0.12	-0.04	-0.02	-0.04
**Acrophase_R-L_**	-0.24	-0.12	-0.24	-0.004
**Mean Activity**	-0.16	0.04	0.02	0.06
**R Activity**	-0.04	0.13	0.03	0.08
**L Activity**	-0.21	-0.03	-0.01	0.03
**Activity_R-L_**	0.22	0.16	0.06	0.10
**ME Survey**	-0.03	0.01	0.02	-0.24

All correlations were non-significant.

### Analysis of Circadian Wrist Activity by Sex and Age

The participants were divided into two age cohorts, based on research demonstrating that circadian patterns change during puberty and young adulthood, with dramatic shifts after the age of twenty [5]. Groups consisted of young adults (ages 16-20) and older adults (ages 21-22). Of the 90 participants, there were 27 young females (18.67 ± 0.31 years), 18 older females (21.11 ± 0.07 years), 21 young males (19.48 ± 0.20 years) and 24 older males (21.29 ± 0.09 years).

**Figure 3:**
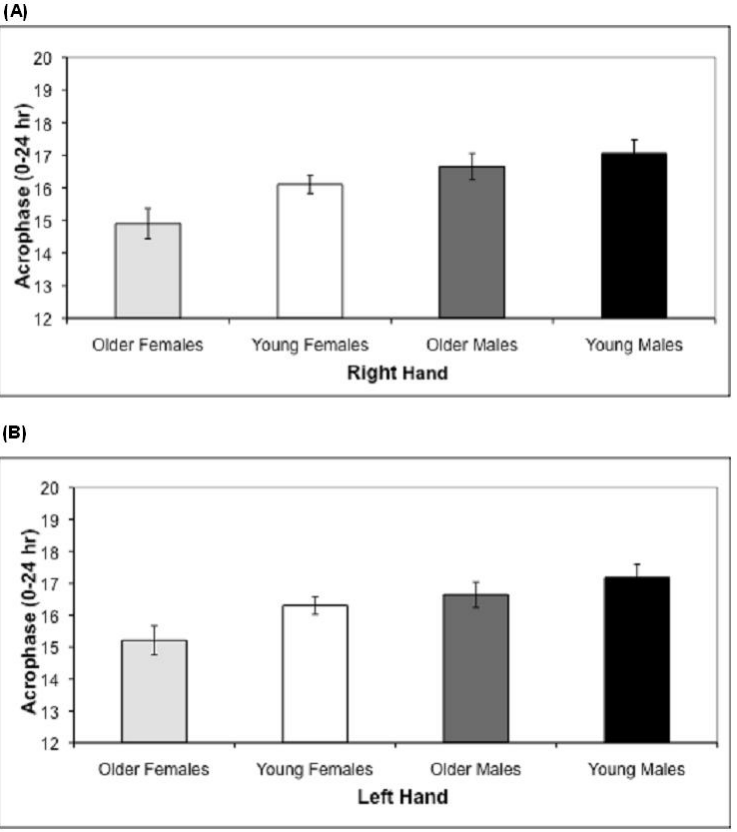
**Right and Left Mean Acrophase for Young vs. Older Male and Female Subjects**. Right (A) and Left (B) mean acrophase (± standard error) for young and older males and females. Males are shown in black (young) and dark gray (older). Females are shown in white (young) and light gray (older). There were main effects for sex (p = 0.002) and age (p = 0.040). The four age and sex groups analyzed show peak activity at different times of the day. The order is as follows ("<" means "peaks before"): older females < young females < older males < young males.

**Figure 4:**
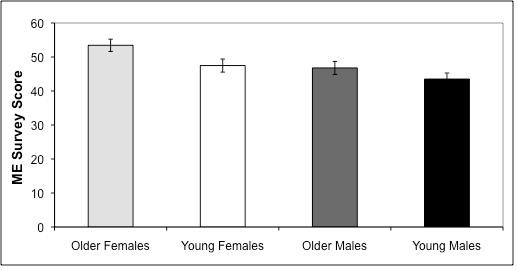
**ME Survey Scores for Young vs. Older Male and Female Subjects**. Morning Evening Preference (ME) scores from the Horne-Ostberg questionnaire for young and older males and females. Males are shown in black (young) and dark gray (older). Females are shown in white (young) and light gray (older). There were main effects for sex (p = 0.02) and age (p = 0.02). The four age and sex groups analyzed show ME scores in the following progression: older females > young females > older males > young males. The higher the ME score, the greater the preference for morning activity.

Acrophase and ME both showed significant main effects for sex and age, without any sex by age interaction effects. Activity for older subjects peaked before young subjects in the right (Figure 3A; p = 0.04) and left hands (Figure 3B; p = 0.03), and older adults showed a preference for morningness in the ME survey while young adults did not (Figure 4; p = 0.02). As seen in the previous analysis by sex alone, female activity peaked earlier than male activity in the right (p = 0.002) and left (p = 0.007) hands (Figure 3), and females showed a preference for morningness in the ME survey while males did not (Figure 4; p = 0.02). Relative positions among age-sex cohorts were identical for right and left acrophase and for ME: young adults demonstrated more male-like patterns of activity while older adults demonstrated more female-like patterns, i.e. young adults had later acrophase and lower ME scores. Young females and older males were most similar.

### Correlation of Digit Length with Measures of Circadian Wrist Activity and Handedness

In order to explore the use of digit length as a putative marker for pubertal androgen exposure [30,34-36], correlations of digit length were run against: activity, acrophase, ME and HHP. Composite averages for 2D and 4D across hands, and an average digit length (D) were calculated and tested by sex. Since males and females have significantly different digit ratios and thus different variation in digit length i.e. less variation for females since ratios are closer to 1.0, 2D and 4D were not averaged within hands. Correlation coefficients for mean 2D, 4D and D are displayed in Table 3. There were no significant correlations between mean digit length and circadian activity or handedness.

**Table 3:** Pearson's correlation coefficient between mean digit length and measures of circadian wrist activity.

	**Males**			**Females**		
	**2D**	**4D**	**D**	**2D**	**4D**	**D**

**Mean Acrophase**	0.15	0.13	0.14	0.08	0.11	0.10
**R Acrophase**	0.14	0.13	0.13	0.12	0.16	0.14
**L Acrophase**	0.16	0.13	0.15	0.04	0.06	0.05
**Acrophase_R-L_**	-0.11	-0.02	-0.07	0.23	0.28	0.27
**Mean Activity**	0.10	0.12	0.11	0.03	0.01	0.02
**R Activity**	0.11	0.08	0.10	0.09	0.06	0.08
**L Activity**	0.07	0.13	0.10	-0.04	-0.04	-0.05
**Activity_R-L_**	0.02	-0.08	-0.03	-0.22	0.16	0.19
**ME Survey**	0.02	0.04	0.03	0.03	0.09	0.06
**HHP**	-0.19	-0.10	-0.15	0.09	0.01	0.05

All correlations were non-significant.

## Discussion

We predicted that patterns of circadian activity would be correlated with 2D:4D if the prenatal androgen exposure that contributes to the development of digit ratio also affects SCN development. Results showed that: 1) 2D:4D was lower for males than for females, with greater differences in the right hand; 2) while total amount of bilateral circadian activity was the same for males and females, patterns of activity were sexually dimorphic such that female activity peaked earlier in the day and females were more morning-type; and 3) there was a robust *lack *of correlation between digit ratio and any measure of circadian activity.

Previous findings for sex and hand differences in 2D:4D were replicated in this study. Mean values for 2D:4D (0.958 ± 0.004 for males and 0.972 ± 0.004 for females) are in the range of those reported for adults [13,15]. Ethnicity and sexual orientation, factors known to correlate with digit ratio [13,16,21,25,29,37], were not accounted for in this study. However, as McFadden and Shubel suggest [17], the effect of ethnicity may be on the value of the digit ratio and may not affect the sex difference, which appears to be relatively stable. The lack of significant sex differences in left 2D:4D in this study parallels previous reports and supports a case for right-sided sexual dimorphisms [17,27,30].

Results demonstrating sex differences in phase of circadian activity [5] and activity preferences [6,7] replicate findings reported elsewhere. Additionally, earlier acrophase for females was correlated with preferences for earlier activity (based on ME survey data) while later acrophase for males corresponded with preferences for activity later in the day, indicating that behavior was strongly correlated with stated preferences and presenting the possibility that ME could accurately substitute for Actiwatch data in certain situations. Of note, only the right acrophase generated a correlation with female ME, while ME was correlated with left and right acrophase in males. Perhaps the ME survey is more sensitive to day/night differences in activity, making it less able to capture variation in female acrophase which occurs within daylight hours. These patterns of relationships warrant further investigation.

Our sample did not compare left- and right-handers but we did find that for combined subjects, activity peaked first in the dominant hand of right-handed individuals and that the right hand was used more; this provides corroboration of work by Natale [2]. Thus overall, the activity data reveal effects for both sex and hand.

The simultaneous action of posterior *HOXA *and *HOXD *genes in the development of the genital bud and limbs has been used to explain the link between androgen and the establishment of digit ratio [38,39]. Androgen is also present during the period of prenatal brain organization [29,40] and if early androgen exposure contributes to the regulation of bilateral circadian activity later in life, digit ratio should be correlated with circadian activity rhythms. However, we found no evidence to support such a relationship; for example, low 2D:4D was not associated with increased mean activity as might be predicted from prior associations of low digit ratio with increased aggression and sensation seeking [18-20]. 2D:4D was not correlated with acrophase (Figure 2), degree of right-handedness, or ME preferences. In fact, no measure of digit ratio was associated with any measure of circadian activity. Thus, we conclude that the prenatal hormone exposure which results in sex and hand differences in digit ratio, is independent from regulatory mechanisms that yield sex and hand differences in circadian wrist activity during adulthood. It is possible, however, that any effect of prenatal androgen is masked or countered by later hormone exposure. Future research could explore the relationship between digit ratio and circadian activity in pre-pubescent individuals.

Sexual dimorphisms in circadian activity have been shown in the diurnal rodent *Octodon degus *and are dependent upon postnatal/pubertal exposure to gonadal hormones [11]. Sex differences in phase and period of degu activity appeared following a delay from puberty, so Hummer and co-workers [41] concluded that circulating hormones are not responsible for the differences in activity, but that gonadal hormones modulate the circadian system, perhaps through androgen receptors in the SCN. Findings from human studies also suggest that sexually dimorphic activity patterns occur post-pubertally and with some delay [5].

Our results revealed that both sex and age had main effects on phase of activity (Figure 3A and 3B) with female and older individuals active earlier in the day. Thus, the relationship of the age-sex cohorts is in the order of old females<young females<old males<young males. The same main effects of sex and age were present for ME preferences (Figure 4).

One possible interpretation of the results is that the phase delay in males (relative to females) represents the lag in puberty for males vs. females, and that the sex differences in phase delay reflect the timing (delay) of puberty for males. It must be noted that the cohorts have significant sex differences in age (p = 0.0004) with females being younger than males. However, since young individuals are more male-like (phase delayed), the fact that females are younger than males increased the difficulty of demonstrating sex differences. Based on our findings, future circadian research using college students should consider age as an important variable since significant differences were found within this narrow age group.

Recently, the relationship between digit ratio and digit length has been investigated as a means of comparing effects of prenatal vs. pubertal androgen: while the ratio of the digits (especially the second to fourth) has become routine as a putative marker for prenatal exposure [13,18,29,31,42], the length of the digits has only recently been used as a proxy for pubertal androgen exposure [30,34-36]. The rationale supplied is that sex differences in finger length appear following puberty due to aromatase actions on androgen in the tissue. One study found modest associations of personality traits with digit length and fewer with 2D:4D [35]. A second study used correlations of 2D:4D and digit length to predict handedness and suggested that release of similar levels of androgen prenatally and pubertally (e.g. low 2D:4D and long digits, corresponding to two rounds of high levels of androgen) could predict right-handedness [30]. Most recently, Bescos et al. [34] explored the relationship of finger length with sport performance in women and Voracek [36] found "spurious" associations of superstitious beliefs and digit length in women.

Subsequent analyses of our data were conducted to explore a possible relationship of digit length with the regulation of bilateral circadian activity. For purposes of comparison with published results [30], we averaged lengths of all four digits (D), and also averaged 2D and 4D across hands. Analysis of 2D and 4D showed predicted sex differences: males had significantly longer digits. However, digit length was not correlated with circadian activity, ME, or degree of handedness. Thus, there is no evidence that the putative markers for prenatal or pubertal androgens are associated with circadian activity and neither marker provides an explanation for sexually dimorphic patterns of activity.

## Conclusion

In summary, digit ratio was lower for males than for females with greater differences in the right hand than the left. Phase of activity was found to be sexually dimorphic and age-dependent. ME preferences correlated with activity for males and females and was also age-dependent. The lack of correlation of circadian activity rhythms with either digit ratio or digit length suggests that the sexually dimorphic regulation of circadian activity is independent from the mechanisms of hormone exposure that affect digit growth. Future research should continue to explore the basis for post-pubertal sexual dimorphisms in phase of activity, especially the relative contributions of prenatal, pubertal and circulating gonadal hormones.

## Competing interests

The authors declare that they have no competing interests.

## Authors' contributions

DBM proposed and designed the study. CR collected the data. CR and DBM analyzed the data, wrote drafts and revised the manuscript. All authors read and approved the final manuscript.
